# Development and challenges of autonomous electric vertical take-off and landing aircraft

**DOI:** 10.1016/j.heliyon.2024.e41055

**Published:** 2024-12-10

**Authors:** Lijuan Hu, Xufei Yan, Ye Yuan

**Affiliations:** a Zhejiang Financial College, Hangzhou, 310018, China; b Tianmushan Laboratory, Hangzhou, Zhejiang Province, 311100, China; c Swansea University, Swansea, Wales, SA2 8PP, UK

**Keywords:** Advanced air mobility, Autonomous eVTOL aircraft, Flight control, Reliability, Airworthiness regulation

## Abstract

Urban Air Transportation (UAT) encompasses private aircraft, air taxis, and specialized missions. These missions include aerial sightseeing, logistics transportation, emergency response, and anti-terrorism operations. They impose stringent requirements on advanced air mobility (AAM) aircraft. These requirements include efficient hovering performance, high-speed cruising capability, and compliance with strict safety and clean energy standards. Consequently, one of the core vehicles for AAM is the efficient and reliable eVTOL (electric vertical take-off and landing) aircraft. Therefore, this paper presents a review of current research on eVTOL aircraft, and highlights potential research paths to advance this innovative field. We begin by classifying and analyzing the latest eVTOL aircraft configurations currently in production, offering an overview of their applications. Subsequently, we delve into key autonomous eVTOL aircraft technologies encompassing electric propulsion, flight control method, sensing & perception, decision-making, and safety & reliability, elucidating recent progress in each domain. Furthermore, we engage in a discourse on the regulatory and societal challenges, including a discussion on airworthiness regulations, that are pertinent to the integration and operation of autonomous eVTOL aircraft. Finally, we conclude by providing future trends and recommendations of autonomous eVTOL aircraft technology, focusing on its interaction with air traffic control system, the adaptation of urban infrastructure, and the design of efficient human-machine interaction protocols.

## 1 Introduction

Urban air transportation, including private flights and special missions like aerial tours, logistics, disaster relief, and security operations, sets high standards for the development of forthcoming urban aerial vehicles. These aircraft need to excel in efficient hovering, high-speed flight, and provide enhanced safety measures while utilizing sustainable energy sources and incorporating innovative features. Conventional helicopters exhibit superior efficiency in vertical take-off and landing (VTOL) operations, hovering maneuvers, and flight at low altitudes and velocities, coupled with unique capabilities for backward and sideward flight. However, their forward speed is limited due to the asymmetric airflow affecting the rotor blades, with the advancing blade's fluid compressibility and the retreating blade's flow separation [1]. Conversely, traditional fixed-wing aircraft offer the benefits of speed and range but struggle with VTOL, hovering, and low-speed flight, which limits its application in the Urban Air Mobility (UAM).

**Fig. 1:**
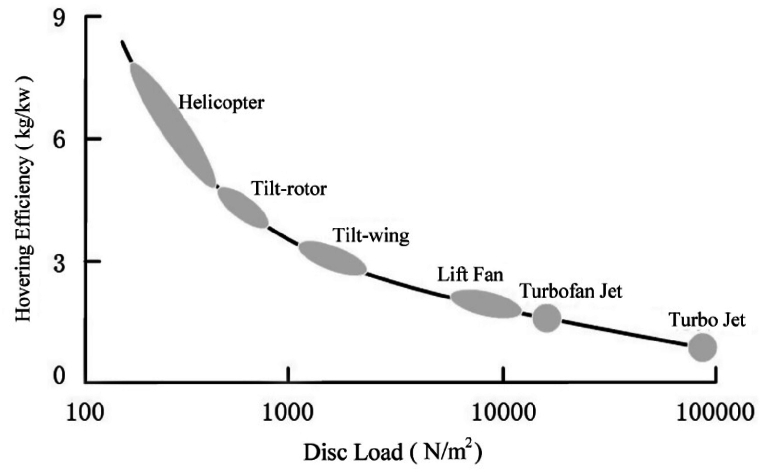
Relationship between hovering efficiency and disc load [2].

Based on extensive research and analytical insights into the field of aerodynamics, it's quite a challenge to design an innovative aircraft that combines a helicopter's efficient hovering with a fixed-wing aircraft's high-speed cruising capabilities, all at a reasonable cost. A critical element in the design is the rotor disc load. Fig. 1 illustrates how hovering efficiency relates to disc load across various vertical take-off and landing (VTOL) vehicles [2]. Aircraft with lower disc loads tend to exhibit better hovering efficiency, similar to traditional helicopters and multi-rotor drones. However, as disc load increases, hovering efficiency decreases while maximum flight speeds increase, as demonstrated by aircraft such as the F-35 B and Harrier fighter [3]. The requirements for high-efficiency VTOL performance and high cruising speed create a trade-off. The design of next-generation aircraft must carefully balance the demands of speed, VTOL capability, fabrication complexity, and economic viability.

In recent years, eVTOL aircraft, which is equipped with DEP (distributed electric propulsion) system, renewable energy, advanced aviation materials, artificial intelligence, and 5G networks, have emerged as the leading unmanned aerial vehicles in advanced air traffic (AAM). As the development of DEP technology, the capacity for sustaining propulsion redundancy is markedly augmented. If a portion of the rotor or propeller fails, the remaining components can ensure the aircraft's safe descent or even enable it to complete its flight mission. Consequently, DEP technology has become a popular choice in the design of advanced eVTOL aircraft.

The rapid development of technology has led to the launch of various eVTOL aircraft, including Ehang 216, Volocity, Joby S4, Lilium Jet, and other models. In the United States, Joby is the leading company in achieving manned operations, having obtained FAA Part 135 operational certification in 2022, with model type certification pending [4]. Following closely behind, Archer is poised to achieve commercial operations by 2025 [5]. In Europe, companies such as Volocopter, Lilium, and Airbus have made significant progress in the development of eVTOL. Notably, Volocopter has constructed a vertiport near Paris and aims to showcase the Volocity 2-seater aircraft during the 2024 Paris Olympics [6]. In China, EHang and Autoflight are actively engaged in the development of eVTOL aircraft. EHang's EH216-S unmanned aerial vehicle has received the world's first Type Certificate in the eVTOL field [7].

Due to the significant advantages of eVTOL aircraft, they have garnered the attention of the U.S. Air Force. In February 2020, the US Air Force launched the ‘Agility Prime’ program to demonstrate and validate eVTOL technology for applications in short-distance transport, special operations, and search and rescue missions [8].

**Fig. 2:**
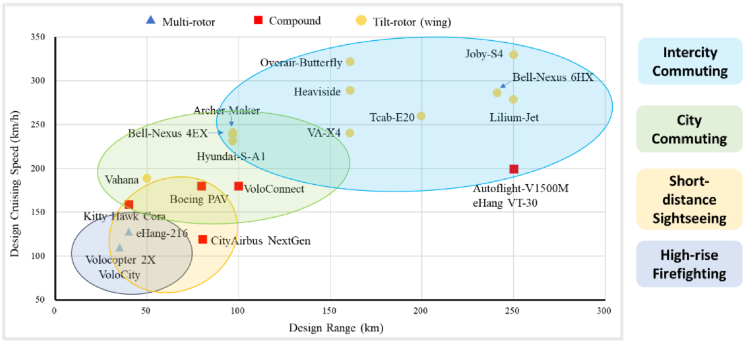
Different kinds of eVTOL aircraft towards different scenarios (The data source is from Refs. [[2], [3], [4], [5], [6], [7], [8], [9]]).

Although still in the early stages of development, eVTOL aircraft have made significant strides toward practical application worldwide. The primary technical configurations employed in eVTOL designs include multi-rotor aircraft, winged compound aircraft, and tilt-rotor (wing) aircraft, as illustrated in Fig. 2 [[2], [3], [4], [5], [6], [7], [8], [9]].

The configurations of eVTOL aircraft do not have absolute advantages or disadvantages, as they are simply dependent on scenarios. It is important to emphasize that the optimal design of configuration should be carefully evaluated based on the development stage and specific application scenarios, weighing its benefits against its drawbacks.

The primary objective of this review article is to offer an accessible and comprehensive overview of the emerging field of eVTOL aircraft to an audience with a foundational understanding of classical aviation concepts, who may not yet be familiar with eVTOL technology. The article aims to serve as an educational resource that clarifies the current state of development and future trajectories of key technologies in the eVTOL sector. By presenting an overview of these technologies, this paper endeavors to highlight advancements, address existing challenges, and project potential future developments in the domain. It is intended to equip readers with the intellectual framework necessary to appreciate the transformative potential of eVTOL aircraft in the context of urban and regional air mobility. In Section 2, we categorize and analyze the latest eVTOL designs in development, along with an overview of their application status. Section 3 focuses on essential technologies for autonomous eVTOL operations, including electric propulsion, flight control, sensing & perception, decision-making, and safety & reliability, while also summarizing regulatory and societal issues. Section 4 discusses emerging trends and provides recommendations, emphasizing the importance of air traffic management, urban infrastructure integration, and the development of human-machine interfaces. Finally, conclusions are drawn in Section 5.

## 2 Description of different types of eVTOL aircraft

### 2.1 Multi-rotor eVTOL aircraft

Multi-rotor eVTOL aircraft typically feature electric rotors arranged symmetrically, offering superior aerodynamic performance during hover and low-speed flight [10]. This design lends these eVTOLs excellent maneuverability and robust hovering capabilities, making them ideal for short to medium-range missions like crop protection, urban aerial firefighting, and sightseeing. The absence of extra components like traditional propellers, wings, or tilt rotors results in a lightweight structure, reduced manufacturing costs, and streamlined control systems. These benefits facilitate their commercialization and application in short-to-medium term initiatives.

Currently, multi-rotor eVTOL aircraft represent the pinnacle of technological maturity within the eVTOL industry. Prominent exemplars consist of designs such as the HEXA, VoloCity, Ehang 216, XPeng AeroHT X2, and ZJCopter-I, as shown in Table 1.

**Table 1:** Representative products of multi-rotor eVTOL aircraft (The data source is from Refs. [[2], [3], [4], [5], [6], [7], [8], [9]], as well as the official website of the corresponding company).

Products	HEXA	VoloCity	EHang216	AeroHT X2	SkyDrive	ZJCopter-Ⅰ
Appearance						
Company	LIFT Aircraft	Volocopter	EHang	XPeng	SkyDrive	Zhejiang Lab
People	1	2	2	2	3	1
Total Weight	310 kg	900 kg	650 kg	560 kg	1400 kg	470 kg
Phase	Flight test	Applying for certification	Applying for certification	Flight test	Production model	Flight test
Duration	15 min	∼30 min	25 min	35 min	30 min	25 min
Range	∼25 km	∼35 km	30 km	unkown	15 km	∼25 km

### 2.2 Winged compound eVTOL aircraft

In recent years, the development of winged compound eVTOL aircraft, which combines a wing and DEP system, has progressed rapidly. The core idea behind this technical solution is to reduce the lift load on the rotors. During periods of hovering and low-velocity flight, the aircraft functions in VTOL configuration. As the flight speed increases, the rotor speed decreases while the wing gradually bears a greater load. At this stage, the propeller system provides the forward thrust [11]. During high-speed flight, it is common for the rotor speed to be reduced to approximately half, at which point the lift rotors contribute to supporting roughly 20 % of the aircraft's weight. This mode of flight is predominantly utilized to achieve increased velocities [12]. Through the reduction of rotor rotational speed and the optimization of the blade profile's angle of attack, winged compound aircraft are capable of deferring the stall of the advancing rotor blade and mitigating the airflow separation from the retreating rotor blade. As a result, this allows for increased flight speeds, broadens the operational range, diminishes vibrations, and improves maneuverability.

Winged compound eVTOL aircraft are generally suitable for medium and long-distance missions, such as city commuting, intercity commuting and so on. The representative products of winged compound eVTOL aircraft are shown in Table 2.

**Table 2:** Representative products of winged compound eVTOL aircraft (The data source is from Refs. [[2], [3], [4], [5], [6], [7], [8], [9]], as well as the official website of the corresponding company).

Products	Matrix 1	Cora (Generation 5)	CityAirbus NextGen	Autoflight V1500M	ALIA	VT-30	VE25 X1
Appearance							
Company	VERTAX	Wisk Aero	Airbus Urban Mobility	Autoflight	Beta Technologies	Ehang	Volant
People	5	2	4 + 1	4	5 + 1	2	4 + 1
Total Weight	unkown	∼800 kg	∼1500 kg	1500 kg	3174 kg	900 kg	∼2000 kg
Phase	In development	Flight test	In development	Flight test	Flight test	Flight test	Full size ground validation
Duration	>1 h	29 min	unkown	∼1 h	2 h	1.6 h	1 h
Range	250 km	100 km	80 km	250 km	460 km	300 km	200 km

### 2.3 Tilt-rotor (wing) eVTOL aircraft

The tilt-rotor (wing) eVTOL aircraft is equipped with tiltable DEP assemblies. When the rotors are vertically up, it is capable of VTOL, hovering, and low-speed flight. As the aircraft reaches a specific speed, the rotors and engine nacelles (or wings) tilt to a horizontal orientation. This conversion lets the wings take on more of the load while easing the burden on the rotors. In this horizontal position, the rotors function as propellers, enabling the aircraft to achieve high-speed flight just like a fixed-wing aircraft [13]. To reconcile the requirements of hovering efficiency and high-speed performance, tilt-rotor aircraft are equipped with rotor blades that incorporate an increased negative twist angle (ranging from −10° for typical rotor blades to −60° for propeller blades). Consequently, although the hovering efficacy of tilt-rotor aircraft is marginally inferior to that of multi-rotor eVTOL aircraft, the tilt-rotor design demonstrates superior performance in vertical flight and high-velocity cruising. One notable example is the Joby S4, which showcases cutting-edge advancements in rotor airfoil design, hub assembly, wing profile, and the optimization of fuselage structural weight. It achieves a maximum flight speed of 322 km/h (89.4 m/s), a maximum range of 241.4 km, and a maximum duration of 1.3 h [14]. Another similar aircraft, the VX4, shares similarities with Wisk and Archer, featuring vectored thrust and a wing-borne, V-tailed hybrid design for lift and cruise. The VX4 boasts a top speed of 321 km/h while providing minimal noise. The company has revealed that the VX4 eVTOL aircraft can achieve noise levels 30 times quieter than equivalent helicopters.

Tilt-rotor (wing) eVTOL aircraft are optimally designed for medium to long-haul missions that require higher speeds, such as urban commuting and intercity travel. Examples of notable tilt-rotor (wing) eVTOL aircraft models are presented in Table 3.

**Table 3:** Representative products of tilt-rotor (wing) eVTOL aircraft (The data source is from Refs. [[4], [5], [6], [7], [8], [9]], as well as the official website of the corresponding company).

Products	Joby S4	Lilium Jet	Wisk Aero Generation 6	PANTALA Concept H	Archer Maker	E20	Vertical Aerospace VX4
Appearance							
Company	Joby Aviation	Lilium GmbH	Wisk Aero	Pantuo	Archer	Tcab Tech	Vertical Aerospace
People	4 + 1	4 + 1	4	4 + 1	2	4 + 1	4 + 1
Total Weight	1815 kg	1500 kg	unknown	unknown	1508 kg	unknown	unknown
Phase	Applying for certification	Applying for certification	Applying for certification	Scaled prototype validation	Flight test	Scaled prototype validation	Flight test
Duration	1.3 h	1 h	∼40 min	∼1 h	∼30min	∼46 min	∼45 min
Range	241.4 km	300 km	144 km	250 km	97 km	200 km	161 km

### 2.4 Summary of current eVTOL aircraft configurations

The following section provides an introduction and analysis of the relevant research on these three technical solutions, aiming to identify potential development directions in the future.

#### 2.4.1 Multi-rotor eVTOL aircraft

The multi-rotor eVTOL aircraft currently represents the most advanced technology in the field. It is characterized by its exceptional maneuverability and precise hovering capabilities, making it ideal for short to medium-range missions. The lack of extra parts like propellers, wings, or tilt rotors results in a lightweight design, reduced production expenses, and a simple control system. These advantages facilitate its commercialization and suitability for short to medium-term projects. However, it is important to note that the multi-rotor eVTOL aircraft faces certain limitations. The maximum flight speed of such aircraft is inherently limited due to the asymmetry in airflow across the rotor's left and right sides during forward flight. This limitation stems from the compressibility effects on the advancing rotor blade and the flow separation phenomena associated with the retreating rotor blade. Consequently, the efficiency of high-speed forward flight is diminished, resulting in a relatively shorter range and flight duration. These factors restrict its application in medium to long-distance missions.

From an industry and market perspective, multi-rotor eVTOL aircraft are acknowledged for their advanced technological maturity within the eVTOL domain. However, these aircraft exhibit constraints in terms of payload capacity and operational range, which consequently delimit their potential application scenarios. Therefore, the technology of the multi-rotor eVTOL aircraft should focus on exploring short-distance application scenarios and strengthening its inherent advantages, that is, enhancing the ability of fixed-point hovering, improving its fault tolerance ability and wind resistance performance [15].

#### 2.4.2 Winged compound eVTOL aircraft

During hover or low-speed forward flight, the flight control strategy of winged compound eVTOL aircraft is essentially similar to that of multi-rotor eVTOL aircraft. For high-speed flight, the rotational speed of the rotors and the angle of attack of the airfoil are systematically reduced. This refinement not only elevates the velocity of flight and extends the operational flight envelope but also significantly attenuates vibration levels and enhances the aircraft's maneuverability. Winged compound eVTOL aircraft are well-suited for medium and long-distance missions. However, there are some technical challenges.

1. During high-speed flight, slowing down the rotors significantly enlarges the reverse flow region, especially when the advance ratio is over 1. This harsh aerodynamic environment poses challenges for the retreating blades [16].
2. The arrangement of the wings and the propulsion system adds to the aircraft's structural weight and can cause increased aerodynamic disruption between the wings and the rotors.

Consequently, further research and development are required to address these challenges, particularly in mitigating aerodynamic interference and reducing additional structural weight. From an industry and market perspective, winged compound eVTOL aircraft are favored by leading companies in traditional aviation due to their higher flight speed, low development risk, and cost-effective nature.

#### 2.4.3 Tilt-rotor (wing) eVTOL aircraft

The tilt-rotor (wing) eVTOL aircraft is a promising solution for improving the high-speed performance of rotorcraft. Although the hovering efficiency of tilt-rotor aircraft is typically lower compared to conventional helicopters, owing to the more pronounced negative twist angle characteristic of their rotor blades, their hovering capabilities can be competitive with or potentially surpass those exhibited by winged compound eVTOL aircraft. This is attributed to the involvement of all power systems during the VTOL procedure. Furthermore, in contrast to compound eVTOL aircraft, the propulsion systems aboard tilt-rotor aircraft are all designed to generate horizontal thrust at high speeds without encountering the challenge of flow reversal. Consequently, the aerodynamic conditions are more stable, allowing for increased flight velocities. Taking these factors into account, this study posits that the tilt-rotor (wing-equipped) eVTOL aircraft, incorporating DEP technology, aligns with the performance benchmarks requisite for next-generation VTOL aircraft, particularly tailored for urban operational contexts. The tilt-rotor (wing) eVTOL aircraft excels in efficient hovering, high-speed flight, greater range, prolonged endurance, and improved safety. Additionally, it brings environmental advantages with its low noise output. These qualities make it a promising route for the evolution of rotorcraft designs moving forward. Nonetheless, several technical hurdles remain to be overcome.

1. The transition mechanism in tilt-rotor aircraft entails alterations in the aircraft's configuration and velocity, which in turn engenders intricate unsteady aerodynamic interactions affecting the rotors, wings, and other structural elements. Moreover, variables like the aircraft's center of mass and inertia undergo nonlinear fluctuations, introducing further inertial influences. As a result, the governance strategy for navigating the transition between the VTOL and high-speed flight modes encounters considerable complexities [17,18].
2. The mechanical design is complex with additional actuating device and auxiliary, and its accuracy and reliability are difficult to guarantee. At the same time, the development risk and cost are also correspondingly increased, requiring a long development cycle and airworthiness certification process.

Therefore, the key technologies of tilt-rotor (wing) eVTOL aircraft should prioritize the development of a dynamic conversion control strategy between VTOL mode and high-speed mode. Furthermore, it is imperative to guarantee a tilting mechanism that is both secure and dependable, while also meeting the stringent criteria for structural lightweight design. From an industry and market perspective, although this configuration presents additional technical challenges, it offers significant benefits in terms of payload capacity and forward flight speed—representing superior operating economics.

#### 2.4.4 Development of DEP technology

Based on extensive research, DEP technology has experienced significant growth in recent years and is now widely employed in cutting-edge rotorcraft designs [19,20].

The integration of winged compound high-speed rotorcraft with DEP technology presents numerous benefits. Lift and thrust systems can operate perpendicular to each other, minimizing the effects of interaction. This leads to a simpler control strategy and improved propulsion redundancy provided by DEP. However, there are notable drawbacks to consider. This technological approach necessitates the deployment of multiple rotors to achieve vertical lift, complemented by additional propellers to generate horizontal thrust. This configuration leads to an increased weight ratio for the propulsion system and its ancillary structural components. Furthermore, during high-speed forward flight, the rotation speed of the lift rotor decreases, leading to the expansion of the reverse flow region. This challenging aerodynamic setting impacts the rotors' dynamic properties, the aircraft's handling, and its overall stability. As a result, enhancements in forward velocity are considerably restricted.

**Fig. 3:**
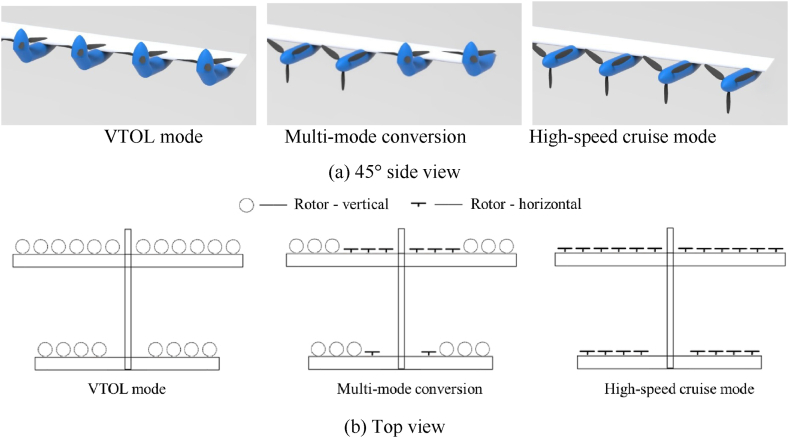
DEP tilt-rotor aircraft can perform multi-mode conversion between VTOL mode and high-speed cruise mode: (a) 45° side view, (b) Top view.

The integration of tilt-rotor aircraft design with DEP technology has facilitated the emergence of tilt-multi-rotor aircraft. This innovative integration markedly augments the aerodynamic performance of rotorcraft across the spectrum of flight velocities, ranging from low to high speeds. In addition, the utilization of DEP technology enables the step-wise tilting of multiple rotors, allowing for gradual increase in flight speed from hovering, while maintaining lift in coordination with the wings, ultimately leading to high-speed cruising. Various stepwise tilting schemes can be employed at different flight speeds to enhance the efficiency of flight missions. With an increasing number of rotors, the available multi-mode conversion strategies become more diverse, as illustrated in Fig. 3.

Nevertheless, the coordinated control process of the lift system during the multi-mode conversion phase of DEP tilt-rotor aircraft is considerably complex. This complexity manifests in two main aspects: (1) During the multi-mode conversion process, dynamic distortions are generated by rotor wakes, resulting in complex unsteady aerodynamic effects on wing impact and aerodynamic interference between rotors, which significantly couples the lift system. (2) The changing aircraft configuration and velocity throughout the multi-mode conversion introduce complexities in flight mechanics variations, control strategy transitions, and control allocation issues. Traditional control strategies and design methods face challenges in achieving autonomous flight control under all modes. Thus, multi-mode conversion coordinated control represents one of the core technologies for DEP tilt-rotor aircraft.

From the perspective of future development, this paper posits that the tilt-multi-rotor aircraft, integrated with DEP technology, aligns with the performance criteria for the forthcoming generation of VTOL aircraft, particularly for deployment in urban environments. The tilt-multi-rotor aircraft presents a suite of advantageous attributes, including efficient hovering performance, the capacity for high-speed cruising, augmented operational range, prolonged endurance, and an enhanced safety profile. Moreover, it exhibits environmental friendliness, low noise emissions, and other advantageous features. Thus, the tilt-multi-rotor aircraft represents a promising direction for the development of novel rotorcraft configurations in the future.

## 3 Key technologies and challenges

**Fig. 4:**
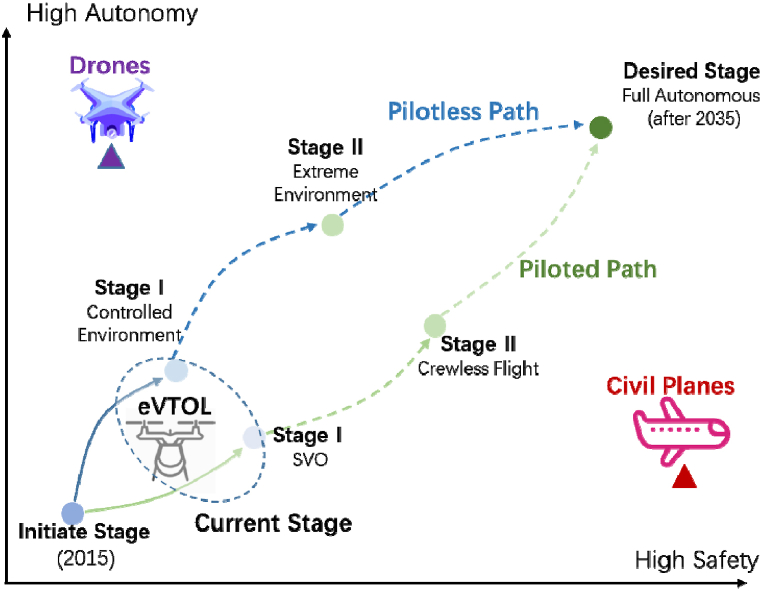
Development trend of autonomous eVTOL aircraft [21].

Numerous studies and media reports have consistently indicated that the prevailing industry viewpoint suggests the future of eVTOL aircraft operations will be characterized by autonomy. However, achieving complete autonomy is subject to several limitations, including aircraft airworthiness, public acceptance, technological maturity, and commercial feasibility. Based on the research and literature described by Xiang et al. (2023), it is evident that two distinct approaches have emerged with the aim of realizing this goal [21]. As depicted in Fig. 4, the first approach, known as the “*piloted path*,” involves a gradual transition from manned piloting to fully autonomous flight. Currently, the primary focus is on creating additional or improved flight technologies to facilitate Simplified Vehicle Operation (SVO) for eVTOL aircraft [22]. Subsequently, crewless flight will be implemented, while retaining pilots for oversight and emergency intervention. Ultimately, the goal is to phase out pilot involvement entirely for full autonomy. This strategy is designed to promotes early-stage airworthiness certification and commercial application, with entities like Joby, Vertical, and Lilium having adopted this developmental trajectory. The second strategy, termed the “*pilotless path*”, prioritizes the development and certification of autonomous flight from the start. It initially targets unmanned flight in controlled settings, with later stages focusing on extending autonomous capabilities to extreme conditions, aiming for broad environmental applicability. Although this approach faces more significant challenges in certification and widespread implementation at the outset, it holds the potential to be the zenith of autonomous eVTOL technology. Companies such as Wisk and E-Hang are committed to this path. However, it's crucial to recognize that both approaches are still in their early development phases.

**Fig. 5:**
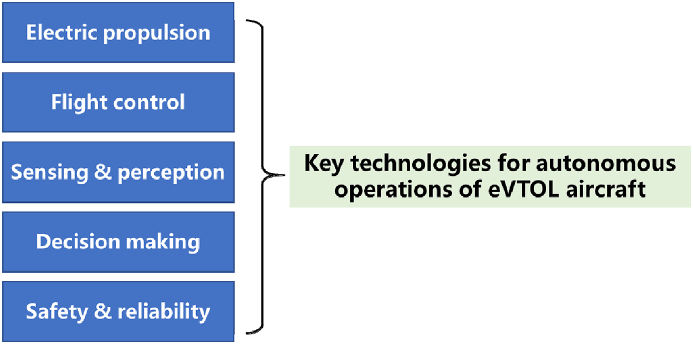
Five key technologies for autonomous operations of eVTOL aircraft.

eVTOL aircraft is an interdisciplinary engineering field that integrates a set of essential technologies for successfully performing autonomous flight. Key technologies include electric propulsion, flight control method, sensing & perception, decision making, and safety & reliability [21,22], as shown in Fig. 5.

Given the limited research focused on applying key technologies for eVTOL aircraft, it is reasonable to draw upon findings from the UAV (Unmanned Aerial Vehicles) domain. These insights could be adapted and utilized for eVTOL aircraft in the near future.

### 3.1 Electric propulsion

The power system of eVTOL aircraft utilizes fully electrified electric propulsion technology, transforming the architecture of aircraft power systems from the energy source. This represents a new direction and a more advanced stage in the development of aviation electrification. Electric propulsion technology utilizes electrical energy as a primary or supplementary power source for the system [23]. This includes hybrid-electric power, batteries, and fuel cells, which drive lift and propulsion devices through electric motors. Additionally, it optimizes energy utilization efficiency through advanced energy management [24], effectively reducing flight noise and pollutant emissions [25,26]. Additionally, the weak sensitivity of the electric power system's power characteristics to atmospheric pressure can significantly enhance its adaptability at high altitudes, demonstrating greater potential for the plateau applicability of eVTOL aircraft.

#### 3.1.1 Battery technology

The technical level of batteries and their various performance indicators are directly related to the performance of eVTOL aircraft. Lithium-ion batteries, due to their higher specific energy, good cycle stability, low self-discharge, no memory effect, and environmental friendliness, are the most promising energy storage equipment in the field of electric aviation. In recent years, the majority of eVTOL aircraft, including the Joby S4, the Archer Midnight, the Vertical X4, the Autoflight V1500M, and the E20 from Tcab Tech, have all adopted lithium batteries as their power source.

**Fig. 6:**
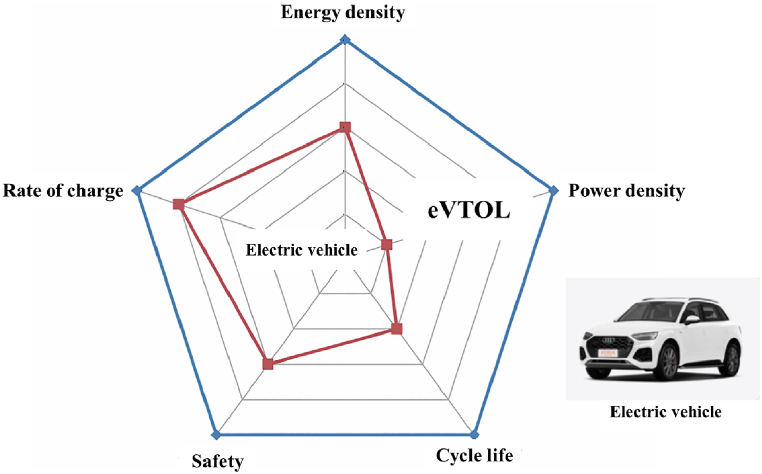
Li⁃ion batteries performance requirements between eVTOL and electric vehicle.

eVTOL aircraft have set higher performance requirements for lithium-ion batteries. The current state-of-the-art energy density of individual battery cells is around 300 W h/kg, and the energy density of battery packs is approximately 220 W h/kg, which is significantly lower than the specific energy of aviation fuel and barely meets the short-range flight needs of small all-electric aircraft [27]. The unique operational profiles and mission cycles of eVTOL aircraft, as well as the demanding operating environments, have put forward higher demands on lithium-ion battery systems [28]. Fig. 6 compares the performance requirements of lithium-ion batteries for eVTOL aircraft and electric vehicles. It is evident that to meet the performance indicators of eVTOL aircraft, it is necessary to comprehensively enhance the energy density, power density, safety, and cycle life of the battery systems.

**Fig. 7:**
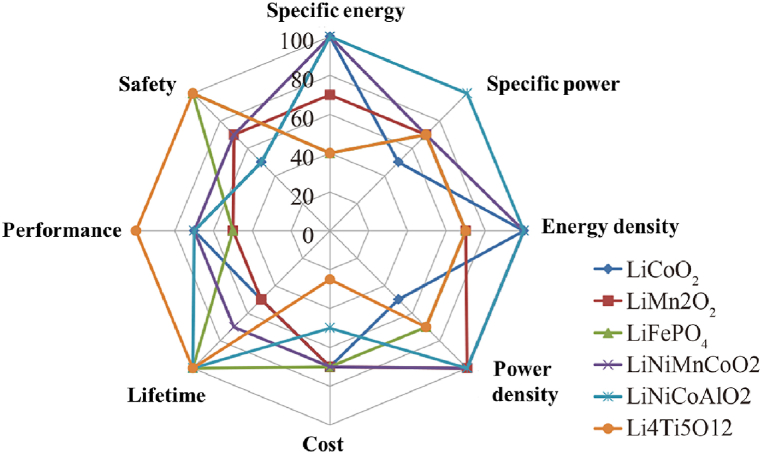
Comparison of performance of current main⁃ stream Li-ion batteries.

Since Sony commercialized lithium-ion batteries with LCO (LiCoO2) cathodes and graphite anodes in the 1990s, the annual growth rate of the energy density of mainstream chemical system lithium-ion batteries has been about 3 %, and since 2000, the annual growth rate has been 4 %. Over the past decade, several other cathode materials (such as NCM, NCA, and LFP) have gradually been commercialized, increasing the energy density of lithium-ion batteries by nearly three times. Fig. 7 presents a comparison of the performance of current mainstream chemical system lithium-ion batteries. Overall, the ternary NCA (LiNiCoAlO2) cells have the best energy and power performance but are more expensive and have the lowest safety; LFP (LiFePO4) cells have the highest safety but only half the energy density of ternary NCA and NCM (LiNiMnCoO2) cells; in comparison, ternary NCM (LiNiMnCoO2) cells have the best overall performance, which also makes ternary NCM batteries the most widely used batteries in current eVTOL aircraft.

Solid-state batteries, with their high energy density and good safety, have become a hot topic of research. In May 2023, Contemporary Amperex Technology Co., Limited (CATL) developed a solid-state lithium-ion battery that achieved an energy density of 500 W h/kg in the laboratory. NASA announced that its battery research department, SABERS, has developed a new aviation-grade solid-state lithium-ion battery with an energy density of 500 W h/kg, capable of operating continuously at high temperatures. However, current solid-state batteries still face issues such as low conductivity of solid electrolytes, low ion transfer capability at the electrode interface, lithium dendrite short circuits caused by solid electrolyte cracks, and safety concerns with metallic lithium anodes. Additionally, current solid-state batteries can only discharge at a very low rate, which is still a significant gap from commercial use.

To meet the extreme working environment requirements of eVTOL aircraft, the performance and tolerance of lithium-ion batteries need to be further enhanced in terms of energy density, power density, portability, and safety. Therefore, it is foreseeable that more and more research will be dedicated to improving the comprehensive performance of lithium-ion batteries [29]. The main current solutions include: ① Improving the key materials and structures of the battery to enhance the stability of the lithium-ion battery itself, such as adding electrolyte additives, improving separator design and manufacturing processes, and designing the structure of cathode and anode materials and solid electrolytes [30]; ② Integrating the battery with the airframe to further enhance the structural and pack efficiency, thereby increasing the system energy density [31]; ③ Designing a battery management system to operate lithium-ion batteries within a suitable and stable working window, monitoring parameters such as current, voltage, resistance, pressure changes, and gas generation to provide early warning and intervention for thermal runaway risks; ④ Designing a battery thermal management system to operate lithium-ion batteries within an ideal temperature range (25–40 °C) [[32], [33], [34]], and at the same time, developing efficient flame retardants or fire extinguishers and designing fire protection systems in response to the characteristics of thermal runaway in lithium-ion batteries that can lead to fires and combustion.

#### 3.1.2 Motor and electronic control technology

**Fig. 8:**
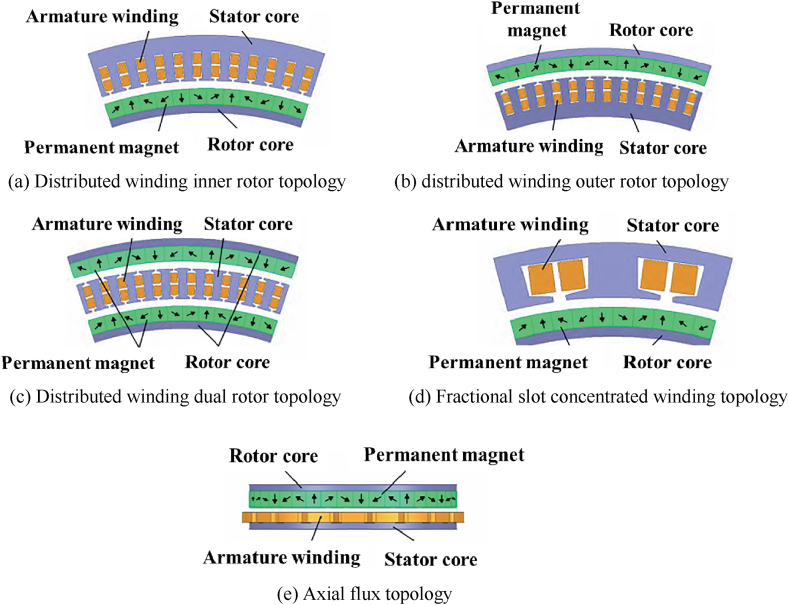
Topology of permanent magnet synchronous motor: (a) Distributed winding inner rotor topology, (b) distributed winding outer rotor topology, (c) Distributed winding dual rotor topology, (d) Fractional slot concentrated winding topology, (e) Axial flux topology.

The electric motor system, as the core power unit in the electric propulsion system, mainly includes the electric motor and the motor driver, directly determining the energy utilization rate and propulsion efficiency of the propulsion system. eVTOL aircraft have high requirements for motor efficiency and torque density, and permanent magnet synchronous motors are a very promising solution for electric propulsion power systems [35]. Current eVTOL aircraft, such as Joby S4, Archer Midnight, etc., all use permanent magnet synchronous motors. Based on the direction of the magnetic field, permanent magnet synchronous motors have two main types: radial flux and axial field [36]. Radial flux and axial system permanent magnet synchronous motors with different topologies are shown in Fig. 8.

Axial flux permanent magnet motors utilize radial space efficiently, offering advantages in power density and torque density, particularly in applications with a relatively small length-to-diameter ratio. Since the power per unit radial length of axial flux permanent magnet motors decreases from the outside to the inside, while the power per unit axial length of radial flux permanent magnet motors is uniform, radial flux permanent magnet motors have more power advantages under the same air gap area and the same maximum rotor tip speed. Limited by the rotor tip speed, axial flux permanent magnet synchronous motors are often used in direct-drive propulsion motor systems ranging from tens of kilowatts to hundreds of kilowatts.

Motor controllers are primarily used to regulate the speed and torque of propulsion motors. Their control response accuracy directly impacts the thrust control accuracy of the aircraft. To improve the control accuracy of motor controllers, experts and scholars have conducted modeling analyses and proposed numerous optimization measures. These measures address dynamic tracking performance, anti-interference capabilities, high-speed low carrier ratios, and high-power low switching frequency driving. In recent years, to enable high-frequency operation of inverters under high voltage and high power in electric aircraft motor systems, a new generation of wide bandgap power devices has gradually replaced traditional power devices. For example, the inverters of the electric propulsion systems of GE and Boeing have adopted SiC power devices [37,38], while the inverter designed by the University of Arkansas has adopted a hybrid module of Si IGBT/SiC MOSFET [39,40]. At the same time, to address high voltage levels and large currents, the high-power inverter in the motor system employs a multi-level topology to reduce voltage stress on individual power devices. This approach not only alleviates voltage stress but also facilitates an increase in switching frequency, while simultaneously reducing output voltage harmonics and, consequently, output current harmonics.

Currently, research on motors and motor drivers for eVTOL aircraft is still in its early stages. To meet the stringent performance requirements of the aircraft's electric propulsion system, urgent technological innovation and breakthroughs are necessary. New types of motor materials, advanced manufacturing processes, and new motor topological structures are key to improving the power density, torque density, efficiency, and reliability of propulsion motors. High-power high-temperature-resistant power modules and intelligent, highly robust motor control technology are important development directions for motor controllers. The physical integration of controllers and motors, comprehensive optimization, rotor-motor integrated design, and efficient thermal management technologies are essential foundations for developing highly integrated and intelligent electric propulsion systems. Addressing these challenges is crucial for engineering advancements in this field.

In summary, lithium-ion batteries represent an ideal choice for eVTOL aircraft due to their high energy density and stability; however, further enhancements in energy density, power density, and safety are necessary. These improvements can be achieved by advancing materials and structures, integrating a battery management system (BMS), and optimizing thermal management. In motor and electronic control technologies, permanent magnet synchronous motors serve as the primary power units, paired with multilevel topology high-voltage, high-current inverters to enhance efficiency and minimize harmonic distortion. To satisfy the stringent performance requirements of eVTOL aircraft, innovations in motor materials and manufacturing processes are crucial, along with the development of highly integrated designs for controllers and motors, and efficient thermal management solutions.

### 3.2 Flight control

Unlike traditional single-rotor helicopters, eVTOL aircraft employ distributed rotors, leading to a more diverse configuration with increased maneuverability and response variability. This significantly escalates the technological challenges in flight control. Current research on control technology for eVTOL aircraft focuses on redundant control surfaces manipulation and coordinated control, robust control for multiple flight modes, fault-tolerant control, and high-safety flight control system architecture design.

eVTOL aircraft possess redundant controls such as rotor speed, collective pitch, and wing control surfaces [41]. The different control effectiveness, precision, and priority are key factors influencing control allocation [42]. For example, the control of rotor speed and collective pitch in eVTOL aircraft demonstrates unique response profiles [43]. Rotor speed control is characterized by a gradual response with robust stability; however, it can induce significant transient power fluctuations due to the dynamics of the motors. In contrast, collective pitch control facilitates rapid responses, ideal for agile maneuvers. By applying the synchronous control method of rotor speed and collective pitch in eVTOL aircraft, the advantages of rotor speed control in steady-state operation and the collective pitch control in maneuverability can be exerted simultaneously [44], thereby enhancing the overall efficacy of flight control. To tackle control allocation challenges, the current strategies for aircraft with redundant control surfaces encompass direct allocation, chain increment, and optimization-based allocation methods [45]. With a focus on simplifying control, ensuring safety, and improving energy efficiency, eVTOL aircraft may adopt techniques such as the generalized inverse method and quadratic programming to devise optimal control allocation strategies. This approach aims to achieve a comprehensive optimization of control performance, response time, and priority considerations [46,47].

**Fig. 9:**
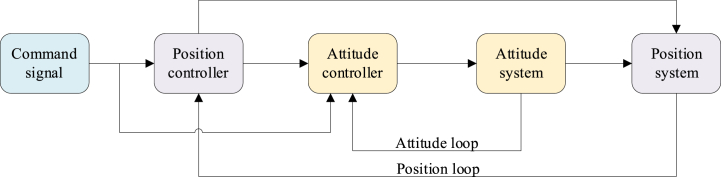
A typical control flow chart of a multi-rotor eVTOL aircraft [21,48].

The flight control system of eVTOL aircraft, integrating advanced hardware and software elements, facilitates autonomous navigation, flight stability maintenance, and execution of critical flight adjustments. Fig. 9 presents the control flow for a multi-rotor eVTOL aircraft. It delineates how desired trajectories translate into command signals, which encompass specified positions, velocities, and attitudes. The system can be divided into two core sub-systems: an outer loop assigned to the governance of positional dynamics and an inner loop allocated to the modulation of attitude control [48].

**Fig. 10:**
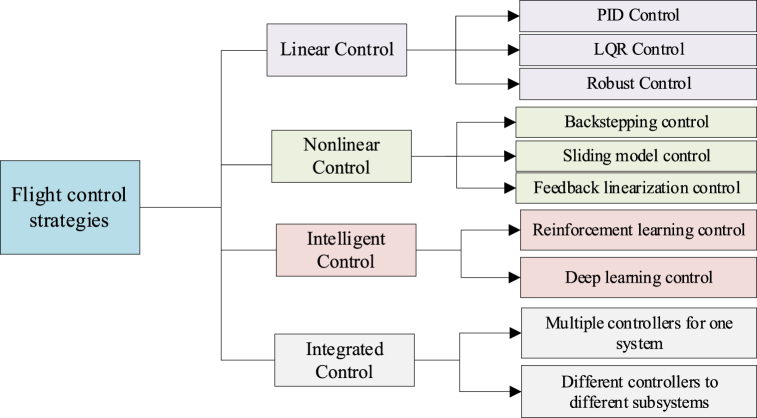
Current control solutions of UAVs [21,[49], [50], [51], [52], [53], [54]].

Fig. 10 provides an overview of the control methodologies utilized for eVTOL aircraft, which can be classified into four main categories: linear control methods, nonlinear control methods, intelligent control methods, and integrated control methods.

**1. Linear control methods.** Linear control methods are predicated on the linearization of a system's dynamics around a nominal operating point [49]. These methods employ mathematical techniques and algorithms to design controllers that proportionally adjust inputs in response to the difference between the actual and target states. Linear control methods have been widely applied in various fields, such as aerospace, robotics, and industrial control systems, due to their simplicity, robustness, and ease of implementation. The Proportional Integral Derivative (PID) Controller [50] epitomizes the ubiquity of linear control, celebrated for its accessible design and versatility. Similarly, Linear Quadratic Optimal (LQR) Controllers [51,52] and Robust Controllers [53,54] have also been extensively used in control applications requiring high accuracy and stability.

**2. Nonlinear control methods.** Nonlinear control methods are designed to address the intricacies of a system's nonlinear dynamics. The primary objective of these methods is to develop controllers capable of handling complex and nonlinear system behaviors, ensuring precise control in nonlinearities and uncertainties. These methods have been increasingly adopted engineering disciplines, particularly in areas where the impact of nonlinear dynamics is pivotal, such as in chemical engineering and biological systems. Examples of these methods include Backstepping controllers [55], Sliding Mode controllers [56], Feedback Linearization controllers [57], Dynamic Inverse controllers [58], and Nonlinear Robust controllers [59]. These approaches offer superior performance compared to linear control methods since they can handle complex and nonlinear systems more effectively.

**3. Intelligent control methods.** Intelligent control methods integrate advanced machine learning algorithms to augment the efficacy of control systems. They capitalize on data-driven techniques to extract insights from historical data and dynamically refine control strategies. By utilizing artificial intelligence and machine learning principles, intelligent control methods effectively manage uncertainties, adjust to dynamic settings, and enhance control performance [49]. Reinforcement learning (RL) control [60,61] and deep learning (DL) control [62,63] are prominent examples of these control methods. RL employs an iterative learning process that fine-tunes controller parameters by optimizing a reward function, whereas DL utilizes artificial neural networks to capture the relationship between system inputs and outputs, facilitating more precise and flexible control mechanisms.

**4. Integrated control methods.** Integrated control methods apply the combination of multiple control approaches or different control methods for various subsystems within an overall system [64]. The integration is designed to improve system performance, augment stability, and facilitate the harmonized management of multiple components. By synergistically merging the advantages of distinct control approaches, integrated control methods aim to enhance control performance. For example, the work cited in Ref. [65] demonstrates swift adaptation to turbulent flight conditions by fusing Proportional Derivative (PD) control paradigms, adaptive control strategies, and deep neural network-based control methodologies, thereby achieving agile flight in challenging windy environments. These methods have been increasingly adopted in various engineering applications, particularly in complex systems where different subsystems require unique control strategies. An example includes autonomous vehicle systems, where various components, such as perception, planning, and control, require different control techniques.

eVTOL aircraft are equipped with redundant control surfaces, and fault-tolerant control is a crucial means to ensure flight safety. Fault-tolerant control involves developing reconfiguration strategies based on the effectiveness of different control surfaces and all-envelope control policies when the aircraft's configuration is altered due to control surface faults [66]. This allows for stable flight by utilizing remaining rotors and control surfaces. Currently, significant research progress has been made in fault-tolerant control mechanisms and stability control using flexible and adaptive methods such as neural networks, multi-model matching, online identification, and adaptive control [67].

To summarize, practical flight control design for eVTOL aircraft faces two primary challenges that need to be addressed in order to enhance the efficacy of these systems. Firstly, accurate establishment of mathematical models presents inherent difficulties due to various factors. These include installation errors, non-perpendicular alignment of multiple motors with the fuselage plane, and vibrations during high-speed rotation. Additionally, asymmetry in axes, inconsistencies in shaft arm length, and mass distribution further complicate the modeling process. The aerodynamic force modeling of eVTOL aircraft is influenced by rotor aerodynamics, wake effects generated by high-speed rotor rotation, as well as external factors such as interference between rotors and between rotors and the airframe. To address these challenges, the utilization of high-precision modeling techniques, including wind tunnels, can prove beneficial.

Secondarily, the eVTOL aircraft represents a quintessential multi-input and multi-output (MIMO) system characterized by nonlinear dynamics and strong inter-component coupling, which is also underactuated. Despite its capacity for spatial maneuverability across six degrees of freedom within a three-dimensional framework—comprising translational displacement along three orthogonal spatial dimensions and rotational articulation about these axes—the eVTOL aircraft is endowed with a limited set of four controllable input variables. These control variables are intricately interconnected with position and attitude control while being susceptible to interference, thereby exacerbating the complexity of control. Overcoming this challenge necessitates the development of sophisticated anti-disturbance and robust control algorithms.

In order to enhance the practical design of flight control systems for eVTOL aircraft, it is imperative to address the challenges of accurate model establishment and effective control strategies.

### 3.3 Sensing & perception

**Fig. 11:**
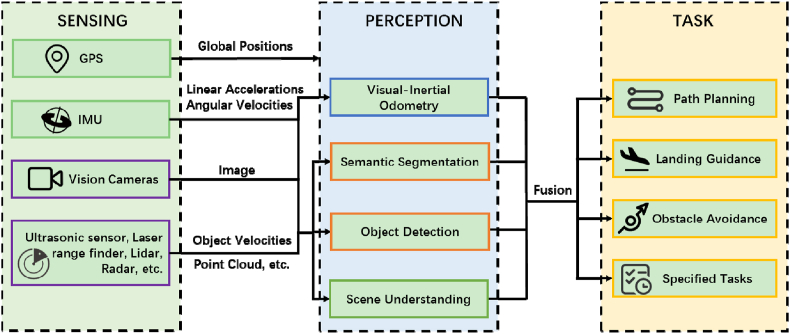
Sensing & perception scheme for eVTOL aircraft [21,68].

The sensing and perception systems of eVTOL aircraft are essential for constructing an accurate digital representation of the operational environment. This model integrates data on objects, events, and situations, along with physical information obtained from both real-time and historical flight data [68]. The dual objectives of these systems are to accurately interpret the surrounding environment, providing detailed information on detected entities, such as their classification, location, velocity, and trajectory, and to produce interpretive semantic data that informs advanced navigation tasks. As depicted in Fig. 11, this includes the capacity to discern spatial relationships among objects, evaluate the viability of landing zones, and detect potential obstructions or dangers.

The efficacy of sensing and perception systems in eVTOL aircraft depends on their capacity to precisely detect and interpret environmental signals in real-time. A variety of technologies have been engineered to fulfill this purpose, including LiDAR, RADAR, and computer vision methodologies. Each of these techniques possess distinct advantages and limitations, which must be carefully considered when designing a sensing and perception system for eVTOL aircraft. For example, LiDAR systems excel at accurately identifying and gauging the distance to objects, rendering them well-suited for tasks requiring obstacle avoidance. Nonetheless, their performance can be compromised in inclement weather, such as during heavy rain or fog. Conversely, RADAR systems exhibit greater resilience to atmospheric conditions but may fall short in discerning intricate object details.

To overcome these challenges, researchers have delved into multimodal sensor fusion strategies, which amalgamate data from various sensors to augment system performance. Additionally, machine learning algorithms have been applied to refine object recognition and classification processes. The advancement of robust and reliable sensing and perception systems is essential for guaranteeing the safe and effective operation of eVTOL aircraft across diverse application scenarios.

1. **Sensing.** The Global Positioning System (GPS) and Inertial Measurement Units (IMUs) are the primary sensors for tracking the motion of eVTOL aircraft. However, GPS can face signal degradation or loss in complex low-altitude environments, and IMUs are prone to drift and bias-related errors [69,70]. To bolster environmental awareness, the integration of a diverse array of sensors into eVTOL systems is essential. Unlike conventional civil aircraft that use ADS-B (Automatic Dependent Surveillance-Broadcast) for surveillance, many smaller aircraft and eVTOLs cannot afford such systems and instead depend on cost-effective sensors like ultrasonic sensors, LiDAR, visual cameras, millimeter-wave radar, and laser range finders for tasks such as obstacle avoidance and altitude regulation. Despite these efforts, challenges remain in accurately determining distances, filtering out false signals, and effectively integrating data from heterogeneous sensors. To address these issues, researchers are investigating advanced techniques. For example, multi-sensor data fusion improves perception accuracy, while Simultaneous Localization and Mapping (SLAM) algorithms aid in autonomous navigation by creating detailed environmental maps and pinpointing the vehicle's location. Machine learning and artificial intelligence algorithms further bolster sensor data processing robustness, leading to more reliable obstacle detection and avoidance. While advancements in sensor technology and algorithm development have been notable, there is ongoing potential for enhancement. The development of innovative sensors with improved range and reduced vulnerability would significantly benefit eVTOL operations. Additionally, integrating real-time sensor data processing and decision-making algorithms into onboard systems could further refine the efficiency and safety of these aircraft.
2. **Perception.** In recent years, there has been notable progress in the automation technologies for small Unmanned Aerial Vehicles (UAVs), encompassing collision avoidance protocols, strategic path planning, autonomous navigation and landing control, high-resolution mapping, and precision positioning systems. Despite these advancements, operating in unstructured environments with dynamic obstacles presents several challenges to ensure the safe and effective functioning of UAVs and eVTOL aircraft. A primary concern is the timely detection of non-cooperative targets to prevent collisions with other aircraft or stationary obstacles like buildings, trees, and power lines [71,72]. Advanced sensing technologies, including radar, thermal imaging, and computer vision algorithms, are being developed to achieve reliable and efficient intrusion detection. Another challenge is maintaining robust and secure flight navigation in environments where satellite signals are unavailable [[73], [74], [75]]. GPS signal disruptions necessitate alternative navigation methods, leading researchers to explore visual-inertial odometry, magnetic field-based navigation, and radio-frequency positioning systems. Safe autonomous landing in the event of a failure is another critical challenge [76,77], involving the identification of suitable landing zones and the execution of a safe landing in unknown conditions. While traditional landings may require pilot assistance, autonomous landing techniques are essential for scenarios where manual control is not an option. Advanced sensors like terrain mapping, LiDAR, and cameras assist in identifying landing areas, while sophisticated control algorithms facilitate precise landing maneuvers. Addressing these challenges involves the development and integration of advanced perception and control technologies into UAV systems. Machine learning can enhance the precision and reliability of intrusion detection and navigation, while reinforcement learning can improve landing control performance. Furthermore, the innovation of new sensor technologies, such as multi-modal fusion sensors and high-precision positioning systems, is expected to significantly improve eVTOL perception capabilities.

In summary, despite the notable advancements in the sensors utilized by eVTOL aircraft, limitations persist in their range of measurement and adaptability to dynamic environments. These constraints can impede the aircraft's ability to identify objects and obstacles from afar, especially in variable environmental conditions. As a result, these limitations may compromise the speed, efficiency, and safety of the aircraft in navigating complex terrains. Furthermore, the sophisticated perception and sensing algorithms essential for the operation of eVTOL aircraft necessitate substantial computational resources. Although computing capabilities have improved, the processing power onboard these aircraft may still be insufficient, constraining the aircraft's capabilities. Developing lightweight and efficient algorithms can alleviate the computational burden on onboard systems, thereby enhancing the aircraft's response time and autonomy. Moreover, the high-precision sensors and computing equipment necessary for advanced perception and sensing are often expensive, which could affect the affordability and widespread adoption of eVTOL aircraft. To mitigate this, researchers are investigating cost-effective and efficient sensor technologies, such as the integration of multiple low-cost sensors to enhance overall perception accuracy. Furthermore, advancements in sensor hardware and software, including the incorporation of artificial intelligence and machine learning, hold the potential to significantly boost the perception capabilities of eVTOL aircraft. For instance, the development of effective data fusion techniques that synthesize information from various sensors can refine the precision of object detection and tracking. In conclusion, while eVTOL perception technologies have come a long way, there remains ample room for further refinement. Addressing the challenges related to range of measurement, adaptability, processing power, and cost will be pivotal in enabling these aircraft to operate in complex environments with greater speed, efficiency, and safety.

### 3.4 Decision making

Autonomous decision-making provides substantial benefits by diminishing the need for human intervention from remote pilots, ground control, and communication networks. Despite these advantages, the prevailing eVTOL aircraft models exhibit a reliance on human directives and lack integrated autonomous functionalities. With the development of eVTOL aircraft field, the necessity for autonomous capabilities becomes more evident to ensure operational safety.

In contrast to military uses where cost is not the primary consideration, the eVTOL industry faces financial constraints that complicate the adoption of costly communication systems, such as satellites and specialized ground infrastructure. This susceptibility to communication breakdowns can disrupt the flow of commands from remote command centers to the eVTOL aircraft, potentially compromising flight safety. In such instances, autonomous decision-making becomes indispensable, allowing the aircraft to independently generate and execute flight instructions without real-time human oversight.

With onboard sensors and computing power, eVTOL aircraft can assess their immediate environment, identify potential threats, and autonomously make informed judgments to guarantee an uninterrupted and safe flight path. This autonomy not only enriches safety measures but also decreases the dependency on constant communication, allowing for reliable performance in adverse conditions or during communication interruptions.

Current research focuses on improving autonomous decision making by developing complex algorithms that fuse sensor data, employ machine learning, and utilize predictive analytics. By integrating data from diverse sensors including cameras, lidar, and radar, eVTOL aircraft gain a holistic understanding of their surroundings, enabling them to make consistent and reliable real-time decisions. Moreover, machine learning facilitates the aircraft's ability to learn from previous encounters, refining its decision-making processes to adapt to dynamic flight scenarios, thereby enhancing overall operational safety and efficiency.

(1) Rule-based decision making

The realization of autonomous operations in eVTOL aircraft can be effectively achieved through the emulation of the cognitive and decision-making faculties inherent to human pilots. Fuzzy logic is proposed as a method to simulate human decision-making ability [78]. This method uses fuzzy logic to establish relationships between key information parameters, including battery status, aircraft functionality, meteorological conditions, and UAV capabilities, enabling the system to make informed decisions on whether to continue or terminate a flight. Complex event processing (CEP) offers another rule-based approach by analyzing event flows and formulating detection rules for autonomous decision-making [79]. Influence diagrams are widely used for decision analysis and uncertainty modeling, providing a framework for optimal actions based on health assessments and robust decision-making under uncertainty [[80], [81], [82], [83]]. The rule-based approach encodes specific scenarios and rules to facilitate decision making. When the encoded conditions are satisfied, the decision-making system responds accordingly. This intuitive and straightforward concept allows for convenient development and optimization. As the number of scenarios increases, additional rules can be added to accommodate them. However, it is important to note that as scenes become more extensive and problems grow in complexity, there is a rapid proliferation of rules, which limits the scalability advantage of rule-based methods.

In addition, rule-based approaches exhibit limited adaptability to uncertain changes due to strict adherence to predefined rules. To address these limitations, researchers are exploring advanced techniques to enhance rule-based decision making. One such approach involves the development of hybrid systems that combine rule-based logic with machine learning algorithms. By integrating machine learning into rule-based decision systems, the ability to adapt to uncertain changes and complex scenarios can be improved. Machine learning algorithms can learn from data and experience, allowing new rules to be discovered or existing rules to be modified based on changing conditions. In addition, the application of optimization algorithms is being explored to reduce the number of rules and improve the efficiency of rule-based systems. By identifying and prioritizing the most critical rules, the expansion of rules can be managed, leading to a more efficient decision-making process.

(2) Data-driven decision making

Data-driven (or learning-based) methods aim to enhance predictive and decision-making models through the utilization of data. These methods are capable of offline training and online reasoning, showing strength and adaptability with new data inputs. This makes them ideal for rapid and agile task planning and decision-making in UAV operations. Recent studies have introduced a variety of data-driven decision-making strategies for UAVs.

For instance, Ref. [84] developed a comprehensive UAV mission planning approach using deep reinforcement learning. This method treats mission planning as a Markov decision process and employs the proximal policy optimization algorithm for model training. Ref. [85] introduced a deep reinforcement learning-based framework for indoor UAV navigation, addressing high-level and low-level planning under uncertain conditions. Such techniques empower UAVs to execute a range of tasks, including surveillance and search and rescue operations [84].

Moreover, some institutions have incorporated GPT (Generative Pre-Trained Transformer) models into UAV decision-making and scheduling, facilitating real-time sensor data analysis and yielding optimized outcomes [86]. The integration of GPT with eVTOL aircraft is seen as a promising avenue for advancing decision-making capabilities. GPT's extensive pre-training allows it to draw from a vast dataset and apply learnings to new situations, enhancing the flexibility of decision-making systems. However, despite the broad applicability of data-driven approaches, they often remain “black boxes”, with their internal mechanisms not always clear, leading to limited interpretability [86]. This lack of clarity can be problematic for safety-critical systems, raising issues related to legality, ethics, and privacy. Moreover, these methods often demand extensive data to fine-tune decision-making systems, which may not always be feasible.

To tackle these issues, researchers are looking into ways to increase the interpretability of data-driven models, such as feature visualization and sensitivity analysis. By understanding the model's inner workings and the features that influence decisions, researchers can improve the transparency and trustworthiness of data-driven decision-making systems.

Additionally, efforts are being made to optimize data usage and reduce the volume of data needed for training. Techniques like transfer learning and meta-learning are being explored to cut down on data requirements by building on knowledge from related tasks or past experiences. These approaches aim to make data-driven decision-making more viable and practical for real-world UAV applications by lessening the data demands.

In summary, autonomous decision-making for eVTOL aircraft hinges on managing two main areas: first, the motion decisions for standard flight operations and specific flight modes, and second, the ability to make emergency decisions when unexpected safety issues arise. Such issues may arise from sensor malfunctions, power system breakdowns, or interactions with non-cooperative entities. Autonomous systems must consider task requirements, the current state of the eVTOL aircraft, and environmental elements to make well-informed choices. This effort requires advances in data integration, processing, and analysis methods. Researchers are delving into various strategies, such as machine learning, deep learning, and reinforcement learning, to create robust decision frameworks. These systems are designed to adapt to changing environments, thus ensuring the safe and efficient operation of eVTOL aircraft. In addition, partnerships between industry leaders, academic institutions and regulators are critical to benchmarking and developing guidelines.

### 3.5 Safety & reliability

Ensuring the high safety and reliability of eVTOL aircraft systems is essential for their successful operation. Safety is the capacity to prevent accidents, while reliability is the capability to operate without malfunctions [87]. Therefore, strategies that enhance system reliability not only maintain functionality but also improve overall safety. Safety design involves a range of activities focused on identifying and managing hazards through comprehensive safety evaluations.

In civil aircraft design, compliance with airworthiness standards is key to establishing safety criteria. Safety requirements are continuously improved throughout the design process, in line with ongoing safety assessments. For instance, the Federal Aviation Administration (FAA) establishes regulations and standards for aircraft certification within the United States, including those for eVTOL aircraft. Table 4 outlines the correlation between hazard categories and the likelihood of faults across various aircraft types, offering a structured approach to evaluating potential risks and their frequency.

**Table 4:**
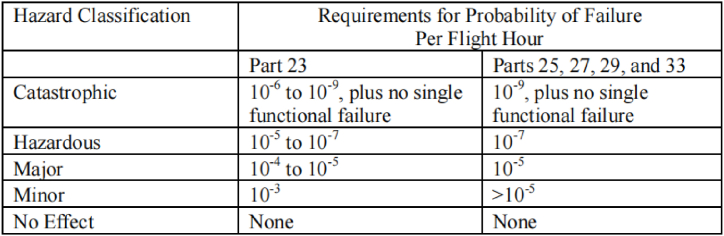
Relationship of hazard categories to probability of failure for different categories of aircraft [88].

**Fig. 12:**
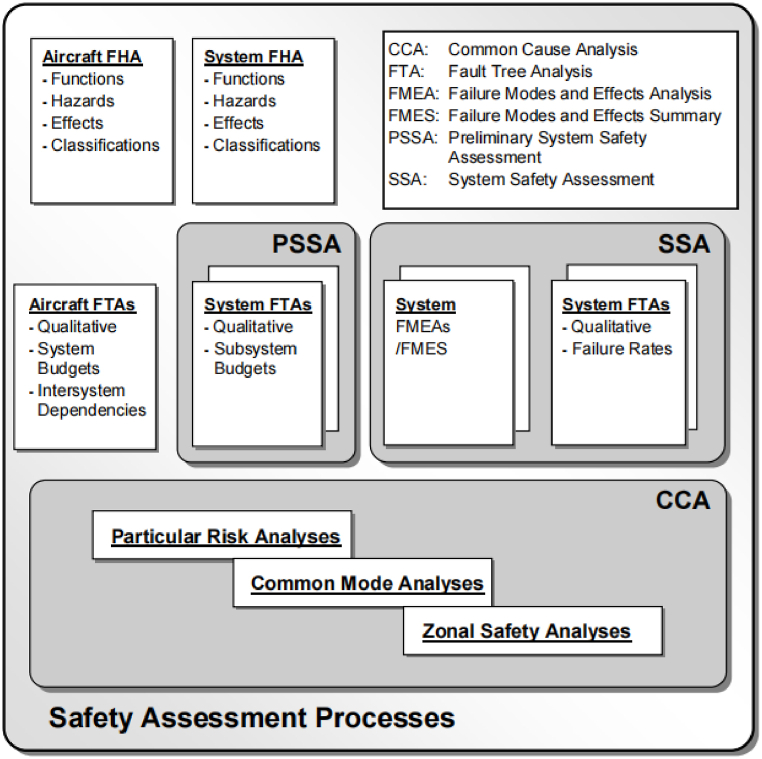
Safety assessment processes from SAE ARP 4761 [88].

Hazard identification and subsequent risk assessment are vital components of the safety evaluation process. Fig. 12, as detailed in SAE ARP 4761 [88], illustrates the safety assessment procedures, which guide the evaluation throughout the aircraft system development. This process comprises four principal stages: defining system requirements, delineating system architecture, conducting system analysis, and performing system verification. These stages facilitate the identification of hazards, the assessment of related risks, and the formulation of strategies to minimize these risks to acceptable thresholds.

Following a thorough safety assessment, it is critical to apply preventive actions to reduce the likelihood of accidents or disasters. In aviation, adopting fault tolerance strategies is essential for managing both internal and external risks inherent in flight operations. Internal risks can include a variety of potential malfunctions such as component failures, flight control issues, actuator problems, sensor malfunctions, communication breakdowns, battery defects, and structural damage. External risks, in contrast, are related to negative environmental factors, obstacles, or potential attacks that may endanger the aircraft's safety.

**Fig. 13:**
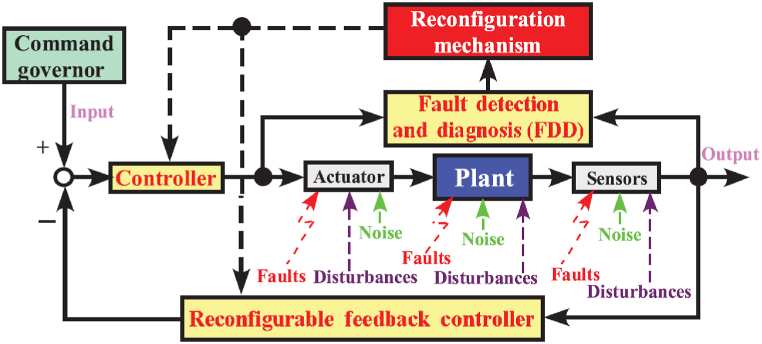
Active fault-tolerant control [89].

To achieve strong fault tolerance, two primary methods are widely used: hardware redundancy and analytical redundancy. Hardware redundancy enhances fault tolerance by duplicating essential components or systems, offering a clear and efficient solution to this challenge. However, it is worth noting that this method comes at a higher cost due to the need for additional hardware resources. In contrast, analytical redundancy achieves fault tolerance through algorithmic means. Among the various forms of analytical redundancy, passive and active fault tolerance are commonly employed methods [89]. Passive fault-tolerant control involves the design of robust controllers tailored to specific fault types, utilizing constant controllers to handle dynamic faults. This approach eliminates the need for fault detection, diagnosis, or system reconfiguration. However, it has limitations regarding its fault tolerance. In contrast, active fault-tolerant control involves system reconfiguration following fault detection and diagnosis. This method ensures that the system continues to perform stably and satisfactorily, as shown in Fig. 13, making it highly effective and a preferred choice in the industry.

**Fig. 14:**
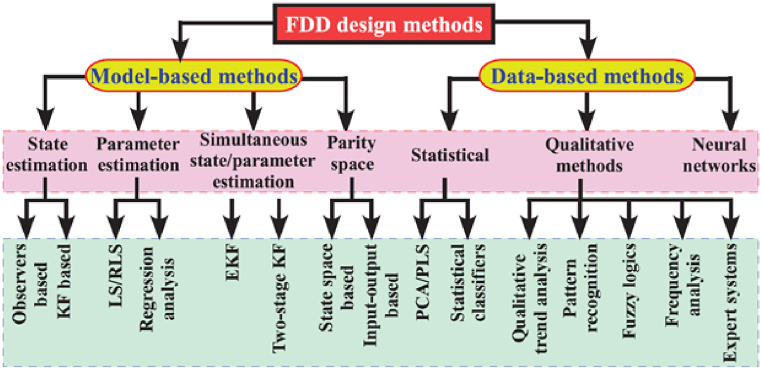
Fault detection and diagnosis method classification [90].

Fault diagnosis methods can generally be classified into two categories: model-based methods and data-based methods, as depicted in Fig. 14 [90].

The model-based fault diagnosis method involves the utilization of mathematical models, known as analytical redundancy, to perform real-time fault diagnosis. However, a limitation of this approach is the inherent challenge of obtaining accurate mathematical models that are applicable to practical scenarios. Alternatively, the data-based fault diagnosis method employs feature extraction techniques from data and identifies faults based on the observed changes in these features. This approach has the benefit of not needing detailed mathematical or physical models. However, it can face challenges in gathering enough training data, which might restrict its effectiveness in handling every possible fault situation. To tackle these challenges, researchers have introduced several techniques to boost the performance of fault diagnosis. For example, hybrid techniques that merge model-based with data-driven methods have been investigated to capitalize on the strengths of both. Moreover, the application of sophisticated machine learning algorithms, including deep learning and artificial neural networks, has been aimed at increasing the precision and dependability of fault diagnosis systems. These innovations are designed to overcome the issues related to model accuracy and data availability, thereby improving the overall efficiency of fault diagnosis strategies.

In summary, the lack of regulatory frameworks for certifying eVTOL aircraft presents a major challenge for the industry. The development of a viable eVTOL aircraft requires a number of technical challenges that should not be underestimated. The pursuit of enhanced safety and reliability inevitably leads to higher costs. The ultimate goal of the eVTOL aircraft is to combine the high safety and reliability standards of traditional aircraft with the cost-effectiveness and autonomous characteristics of UAVs. This integration is intended to promote broad adoption and large-scale implementation. Therefore, there is an urgent need to develop a new type of fault-tolerant control system that can meet the safety and reliability requirements of eVTOL aircraft, while being cost-effective and practical. Such a system is critical to bridging regulatory gaps and streamlining the certification process for these innovative vehicles. By integrating robust fault detection and management strategies, the control system will improve the safety and reliability of eVTOL operations. Furthermore, creating this fault-tolerant control system requires collaboration across disciplines and progress in multiple areas. Experts, including researchers, engineers, and policymakers, must join forces to tackle the intricate issues surrounding eVTOL regulation and control. Additionally, integrating cutting-edge technologies like artificial intelligence and machine learning could significantly improve the fault tolerance of eVTOL aircraft systems.

## 4 Trends and recommendations

As previously mentioned, eVTOL aircraft are key parts of the Advanced Air Mobility (AAM) system, and their autonomous function requires smooth coordination with other system components. In the following discussion, we will investigate the expected trends in the future evolution of autonomous eVTOL aircraft technology, focusing on its interaction with air traffic management systems, urban infrastructural adaptations, and the development of human-machine interaction interfaces.

### 4.1 Regulatory and societal challenges

#### 4.1.1 Regulatory challenges

The certification of eVTOL aircraft poses considerable challenges and risks due to its innovative nature. So far, no eVTOL aircraft has achieved full certification, leading to significant uncertainty about the regulatory landscape. Within the existing framework of initial airworthiness regulations, eVTOL aircraft are not classified under the categories of Normal/Transport Category Airplanes as defined by FAA Part 23/25 or Normal/Transport Category Rotorcraft as outlined in FAA Part 27/29. In response to this gap, the Federal Aviation Administration (FAA) has initiated changes to its certification approach, introducing a “special class” process for powered-lift aircraft models such as Joby Aero JAS4-1 and Archer M001 [91]. Similarly, the European Union Aviation Safety Agency (EASA) is working towards creating a distinct category for eVTOL aircraft, starting with the establishment of general special conditions (SC-VTOL-01 SC for small-category VTOL aircraft). These efforts show a shared understanding of the need for customized regulations within the industry. However, several regulatory issues remain unaddressed. Foremost among these is the lack of clear regulations for eVTOL aircraft type certification, operations, airspace management, and infrastructure requirements. The current regulatory systems do not offer complete guidelines on product certification, operational procedures, responsibilities and rights, safety measures, security protocols, and other essential areas. Bridging these gaps requires the development of formal regulations based on practical experience with special conditions [92].

Additionally, the integration of autonomous technology in eVTOL aircraft introduces further challenges. Regulatory authorities are cautiously receptive to the idea of fully autonomous eVTOL aircraft, as it deviates from the traditional requirement of having at least one crew member on board. While automation has been integrated into general aviation to some extent, the concept of fully autonomous operations is yet to be widely accepted. Nevertheless, the certification of drones with automatic technology has shown greater tolerance, and efforts are underway to explore single-pilot operations supported by virtual co-pilots [93]. The implementation of autonomous eVTOL aircraft also entails significant challenges related to trustworthiness, certification complexity, interpretability limitations, and the constraints of machine learning and deep learning algorithms [94]. These factors collectively pose obstacles to the successful integration of autonomous capabilities into eVTOL aircraft.

Currently, there is no uniform standard for the airworthiness certification of eVTOL aircraft across different national aviation authorities. A comparison of airworthiness regulations from the European Union Aviation Safety Agency (EASA), the Federal Aviation Administration (FAA) of the United States, and the Civil Aviation Administration of China (CAAC) is presented in Table 5 [7,[95], [96], [97]].

**Table 5:** Airworthiness regulations and standards of eVTOL.

Agency	Approval Category	Status of airworthiness regulations and standards
EASA	VTOL category, divided into basic and enhanced classes based on operational scenarios	In July 2019, specialized conditions (SC-VTOL) were issued for small VTOL aircraft (with a maximum of 9 seats and a maximum takeoff weight not exceeding 3175 kg). Multiple MOC SC-VTOL documents providing guidance on airworthiness compliance methods for VTOL aircraft were issued between 2021 and 2022. In April 2021, specialized conditions for electric/hybrid propulsion systems were released. In May 2023, noise technical specifications for electric VTOL aircraft were published.
FAA	Approved by special category in accordance with 21.17 (b)	In 2022, the draft consultation documents for the certification basis of Joby JAS4-1 and Archer M001 were released, referencing the applicable requirements from 23 fixed-wing, 33 engine, and 35 propeller airworthiness standards.
CAAC	Separate certification for manned and unmanned operation, with specific requirements tailored to each category	The “Special Conditions for EH216-S Unmanned Aerial Vehicle System” were published in February 2022. The “Airworthiness Certification Management Procedures for Civil Unmanned Aerial Vehicle Systems” were issued in December 2022. In November 2023, the “Special Conditions for Autoflight V2000CG Unmanned Aerial Vehicle System” were released.

From Table 5, it can be observed that currently only Europe has issued unified standards and conformity methods for small VTOLs, while China and the United States have tailored airworthiness requirements based on the specific characteristics of each eVTOL aircraft model. In the design of the airworthiness certification system for eVTOLs, Europe and the United States follow the traditional airworthiness route for manned aircraft. Leveraging the recent vigorous development of the unmanned aerial vehicle industry and the relatively complete regulatory framework for unmanned aerial vehicle certification, the Civil Aviation Administration of China has incorporated the airworthiness certification of unmanned eVTOLs into the unmanned aerial vehicle review system based on classification management according to operational risk levels.

In conclusion, addressing the regulatory challenges surrounding eVTOL aircraft certification requires interdisciplinary collaboration and concerted efforts from researchers, engineers, and policymakers. Comprehensive regulations must be established to facilitate the safe and widespread adoption of these innovative aircraft, especially autonomous eVTOL aircraft.

#### 4.1.2 Societal challenges

The successful application of autonomous eVTOL aircraft depends greatly on public trust and acceptance. Mitigating concerns regarding safety, noise, and privacy issues is crucial in gaining public trust [98,99]. Currently, there is still a lack of public trust in the reliability of autonomous technology, VTOL operation, and electrical propulsion systems [98]. Public acceptance of this new transportation mode and their readiness to pay for it are key factors to consider. Studies show that while potential users are intrigued by urban air mobility (UAM), they also have concerns about safety, security, noise pollution, and the environment [98]. To encourage broad acceptance, it is important to tackle the factors affecting the customer experience. Noise pollution, for example, is a major issue that must be managed with effective cabin noise mitigation techniques in the aircraft's design.

Moreover, integrating eVTOL aircraft with current transportation systems and reducing environmental disruptions are critical [99]. During the design phase, the safety perception, vehicle motion, noise and vibration levels, environmental effects, and passenger comfort should be prioritized [99]. To build societal confidence, it's essential to communicate the safety features and redundancies of autonomous eVTOL aircraft. Being transparent about the reliability of these technologies can ease public apprehensions. Additionally, engaging with the community through educational initiatives and outward projects can enhance understanding of the advantages and potential societal impacts of eVTOL aircraft.

In summary, societal issues like safety, noise, and privacy are key to the broad acceptance of autonomous eVTOL aircraft. By tackling these issues with open communication, using noise reduction methods, and focusing on passenger comfort and environmental responsibility, we can boost public trust.

### 4.2 Future trends

#### 4.2.1 Integration with air traffic management

Integrating autonomous eVTOL aircraft into current airspace and traffic patterns calls for a robust Air Traffic Management (ATM) strategy. As these aircraft grow more common, there is a pressing demand for the development of automated routing systems capable of rapidly adapting to meteorological and traffic variations. This involves developing advanced communication systems that allow for smooth coordination between eVTOL aircraft, other planes, ATM systems, and ground controllers. Additionally, the establishment of unmanned traffic management (UTM) systems, designed for the real-time tracking and surveillance of all airborne vehicles within the airspace, is essential for ensuring the safe integration of autonomous eVTOL aircraft into the aviation ecosystem. To integrate eVTOL aircraft effectively, it's essential to establish rules and standards for their operation and interaction with other aircraft. Moreover, comprehensive training for pilots and air traffic controllers is needed to maintain safe and efficient operations. With eVTOL technology's emergence, new regulations and standards must address specific challenges like battery limitations, airspace congestion, and emergency procedures. The seamless integration of eVTOL aircraft into the current ATM systems will require joint efforts from regulatory agencies, manufacturers, and operators [100,101].

#### 4.2.2 Integration with urban infrastructure

Integrating eVTOL aircraft into urban infrastructure presents exciting possibilities for transforming city transportation. The creation of designated landing and takeoff zones for eVTOL aircraft in urban settings represents a promising development that can ensure safe and accurate flight operations in crowded cities. Potential locations for these zones include rooftops of buildings, underutilized parking lots, or elevated platforms above bustling streets. Integrating eVTOL operations into the urban fabric may necessitate the formulation of novel regulatory frameworks and the deployment of specialized air traffic control (ATC) systems. They would enable eVTOL aircraft to navigate adeptly around other aerial vehicles, architectural structures, and potential obstacles within densely inhabited urban settings. Moreover, incorporating eVTOL aircraft into public transit networks could enhance commuting efficiency in urban regions. For instance, commuters could travel to designated landing pads from their homes and then connect to public transit. As eVTOL technology progresses, its integration potential with urban infrastructure is expected to grow. For example, eVTOL aircraft could be deployed for emergency medical services or disaster response, cutting response times, and potentially saving lives. To fully realize the benefits of eVTOL aircraft integration with urban infrastructure, a collaborative approach involving regulatory agencies, manufacturers, and urban planners is essential [102].

#### 4.2.3 Human-machine interaction

Human-machine interaction (HMI) is key to enhancing the user experience and encouraging the adoption of autonomous eVTOL aircraft technology. The evolution of HMI will significantly influence the growth of autonomous eVTOL aircraft technology. A notable trend is the inclusion of voice and gesture recognition, allowing passengers to communicate with eVTOL aircraft in a natural and intuitive way. Users can adjust settings like temperature or control entertainment with simple voice commands or hand movements, which can even affect the flight path, improving the overall comfort and convenience of the journey. Moreover, the adoption of augmented virtual reality (AVR) interfaces is increasingly gaining traction. Pilots and passengers alike can now benefit from augmented reality (AR) headsets, which superimpose critical data onto a real-time visual field. This data encompasses flight-specific metrics, meteorological updates, and traffic alerts, thereby enhancing situational awareness and operational insight. This allows pilots to make more informed decisions, boosting safety, while passengers have an engaging and interactive flight experience. As eVTOL technology continues to develop, its integration with HMI is likely to expand, leading to even more intuitive and user-friendly interactions for both passengers and pilots [103].

### 4.3 Recommendations

The progression and implementation of autonomous eVTOL aircraft encounter a multitude of complex challenges that require resolution to ensure their safe and efficacious integration within urban airspace. To advance the implementation of Advanced Air Mobility (AAM) and eVTOL technologies, strong teamwork among researchers, industry professionals, and policymakers is essential. Several successful public-private partnerships and regulatory frameworks have facilitated the development of eVTOL technologies. In the U.S., the FAA collaborates with companies like Joby Aviation under the Urban Air Mobility initiative, creating safety standards and air traffic management systems. The EU's Horizon 2020 program funds projects that partner industry with research institutions while establishing regulations for safety and environmental impact. Singapore's Smart Nation Initiative promotes partnerships with firms like Volocopter, supported by a regulatory framework for testing eVTOL operations. NASA's Advanced Air Mobility campaign involves research collaborations to develop certification criteria, and Japan's Public-Private Council for Future Air Mobility shapes policies to advance urban transport solutions. Additionally, the UK's Future Flight Challenge encourages aerospace collaborations while the Civil Aviation Authority formulates guidelines for safe deployment. The city of Shenzhen, China, has effectively advanced the eVTOL industry through policy support, technological collaboration, pilot projects, and infrastructure development, exemplifying a successful public-private partnership. These initiatives collectively support innovation and ensure compliance in the growing eVTOL sector.

Based on the successful cases mentioned above, we present several recommendations to support the future development and application of autonomous eVTOL aircraft.

**1. Conduct Comprehensive Testing:** Ensuring the safety and dependability of autonomous eVTOL aircraft requires comprehensive testing. This involves stringent assessments in various weather conditions, across different landscapes, and within busy urban settings [104]. Moreover, both simulations and real-world trials offer critical data on system performance and possible failure modes, facilitating ongoing enhancements. The testing should also consider multiple aspects, including noise levels, energy use, and environmental effects, to promote sustainable and responsible operation.

**2. Invest in Cybersecurity:** Given the significant dependence on technology and communication networks, it's crucial to invest in solid cybersecurity defenses. This involves safeguarding against cyberattacks, hacking, and unauthorized access to confidential data [105]. Creating secure communication protocols, implementing encryption techniques, and deploying intrusion detection systems will help maintain the integrity and privacy of data shared between eVTOL aircraft, ground controllers, and traffic management systems. Additionally, setting up all-encompassing cybersecurity policies and regulations will build trust among all parties involved and fortify eVTOL operations against emerging threats.

**3. Establish Interoperability Standards:** Creating interoperability standards is crucial for the smooth integration of eVTOL aircraft into existing transportation networks. This involves developing consistent communication protocols, data exchange standards, and systems for airspace management [106]. By establishing shared interfaces and protocols, eVTOL aircraft can communicate effectively with other aircraft, air traffic control systems, and ground operators, facilitating efficient and safe operations. In addition, interoperability standards should also consider how eVTOL operations fit into current air traffic control settings and ensure they are compatible with new technologies such as unmanned aerial systems.

**4. Assess Economic Feasibility:** The financial success of eVTOL aircraft is crucial for their broad acceptance. Research should focus on creating affordable propulsion systems, efficient manufacturing methods, and straightforward maintenance plans to guarantee sustainable use over time [107]. Moreover, looking into new business approaches like shared rides and freight services can enhance the economic viability of eVTOL operations. Also, economic evaluations should include considerations of initial infrastructure expenses, energy use, and possible societal advantages to fully grasp the economic implications of eVTOL technology.

**5. Focus on Workforce Training:** As the eVTOL industry grows, there will be a demand for skilled professionals. It's essential that industry and policy leaders invest in training programs to ensure a ready supply of skilled workers for the future needs of eVTOL technology [108]. This involves educating pilots, technicians, and air traffic controllers with the unique expertise required for eVTOL operations. Moreover, building partnerships among educational institutions, businesses, and training centers can promote the sharing of knowledge and speed up the acquisition of skills in critical areas like autonomous systems, electronics, and airspace coordination.

By following these recommendations, the research, industry, and policy sectors can work together to improve autonomous eVTOL aircraft technology and help it become part of city skies. This will lead to the full use of Advanced Air Mobility. Working together with different groups is crucial for facing challenges and making a safe, effective, and lasting eVTOL environment. Also, regularly checking and assessing the use of these ideas will help make things better and make sure the eVTOL aircraft technology stays up-to-date with changes in society, the environment, and technology.

## 5 Conclusion

The article aims to serve as an educational resource that clarifies the current state of development and future trajectories of key technologies in the eVTOL sector, specifically focusing on electric propulsion, flight control method, sensing & perception, decision making, and safety & reliability. It is important to note that this article omits certain topics, such as ground infrastructure and operational aspects. This is because we aim to focus on the core technologies central to current eVTOL aircraft. Our goal is to provide depth in these areas without overwhelming newcomers with the complexities of the entire ecosystem. We recognize the significance of these considerations and will address them in future work.

By presenting an overview of the current state of these technologies, this paper endeavors to highlight advancements, address existing challenges, and project potential future trajectories in the domain. Through the analysis presented, several conclusions can be drawn:

1. Electric Propulsion Systems: Lithium-ion batteries, noted for their high energy and power density, are the preferred choice for eVTOL aircraft, yet there is a need for ongoing improvements in energy density, power density, and safety. These can be attained through material innovation, battery management system integration, and thermal management optimization. For propulsion, permanent magnet synchronous motors, integrated with advanced multi-level inverters, enhance efficiency and reduce harmonics. To meet eVTOL performance criteria, innovations in motor material technology and the adoption of compact, thermally efficient designs for both motors and controllers are crucial.
2. Flight Control Methodologies: Practical flight control design for eVTOL aircraft faces two key challenges. First, accurately establishing mathematical models is difficult due to factors such as installation errors, misalignment of motors, and aerodynamic influences from rotor dynamics and interactions with the airframe. High-precision modeling techniques, such as wind tunnel tests and system identification, can help mitigate these issues. Second, eVTOL aircraft operate as complex multi-input and multi-output (MIMO) systems with nonlinear dynamics and strong inter-component coupling, yet they are underactuated with only four controllable inputs. This complexity necessitates the development of advanced anti-disturbance and robust control algorithms to effectively manage position and attitude control. Addressing these challenges is crucial for improving flight control systems in eVTOL aircraft.
3. Sensing and Perception: eVTOL aircraft have made significant strides in sensor technology, yet they still face limitations in measurement range and adaptability to dynamic environments, hindering their ability to detect obstacles effectively. These challenges may compromise flight speed, efficiency, and safety in complex terrains. Additionally, the sophisticated algorithms needed for perception require substantial computational resources, which may be insufficient onboard. Developing lightweight algorithms can improve response times and autonomy. High-precision sensors are often costly, prompting research into cost-effective solutions, such as integrating multiple low-cost sensors and utilizing AI for better data fusion. Overall, addressing these challenges is crucial for enhancing eVTOL capabilities in complex environments.
4. Decision Making: Autonomous decision-making for eVTOL aircraft focuses on two key areas: managing standard flight operations and making emergency decisions during unexpected safety issues. These systems must integrate task requirements, aircraft status, and environmental factors to ensure informed choices, necessitating advances in data processing and analysis. Researchers are exploring strategies like machine learning and reinforcement learning to develop adaptive decision frameworks that enhance safety and efficiency. Furthermore, collaboration among industry leaders, academic institutions, and regulators is essential for establishing benchmarks and guidelines.
5. Safety and Reliability: The absence of regulatory frameworks for certifying eVTOL aircraft poses a significant challenge for the industry, as developing these vehicles involves complex technical issues that can increase costs. The ultimate goal is to merge the safety and reliability of traditional aircraft with the cost-effectiveness and autonomy of UAVs to facilitate widespread adoption. This necessitates the urgent creation of a fault-tolerant control system that meets safety requirements while remaining practical and economical. Such a system is crucial for bridging regulatory gaps and enhancing certification processes. Achieving this will require interdisciplinary collaboration among researchers, engineers, and policymakers, along with the integration of advanced technologies like artificial intelligence and machine learning to improve fault tolerance in eVTOL systems.
6. Regulatory and Societal Challenges: The regulatory landscape for eVTOL aircraft is evolving, with the FAA and EASA developing specialized certification processes. Key challenges include establishing comprehensive guidelines for type certification, operations, airspace management, and infrastructure. Additionally, societal acceptance hinges on addressing public concerns about safety, noise, and environmental impact through effective communication and advanced noise reduction technologies.
7. Future Outlook: Future trends in eVTOL technology highlight the integration of advanced air traffic management systems, focusing on automated routing and robust communication networks to facilitate seamless coordination among eVTOLs, other aircraft, and ground control. The implementation of unmanned traffic management (UTM) systems will be essential for real-time tracking and management of eVTOL flights, thereby enhancing safety and operational efficiency while addressing public concerns.

In conclusion, while eVTOL aircraft technology has made remarkable progress, there are still substantial hurdles to overcome. A concerted, multidisciplinary effort is required to address the remaining technical challenges and to build the necessary public trust and regulatory frameworks for the successful integration of autonomous eVTOL aircraft. This review aims to contribute to this discourse and to stimulate further advancements in this rapidly evolving field.

## CRediT authorship contribution statement

**Lijuan Hu:** Writing – original draft. **Xufei Yan:** Writing – review & editing, Conceptualization. **Ye Yuan:** Writing – review & editing, Supervision.

## Data availability statement

Research-related Data is not stored in publicly available repositories, and Data will be made available on request.

## Ethics declarations

Review and/or approval by an ethics committee is not needed for this study because we don't work with humans or animals.

## Funding

This research is supported by the 10.13039/501100001809National Natural Science Foundation of China (No. 12202406).

## Declaration of competing interest

The authors declare that they have no known competing financial interests or personal relationships that could have appeared to influence the work reported in this paper.
